# The Effects of Diet Intervention on the Gut Microbiota in Type 2 Diabetes Mellitus: A Systematic Review

**DOI:** 10.7759/cureus.56737

**Published:** 2024-03-22

**Authors:** Kiana Farahbod, Ethan Slouha, Andrew Gerts, Atbeen Rezazadah, Lucy A Clunes, Theofanis F Kollias

**Affiliations:** 1 Department of Pharmacology, St. George's University School of Medicine, St. George's, GRD; 2 Department Microbiology, Immunology, and Pharmacology, St. George's University School of Medicine, St. George's, GRD

**Keywords:** exercise, gut microbiota, nutrition, diet, diabetes

## Abstract

The GI tract hosts a dynamic community known as the gut microbiota, which encompasses thriving bacteria that actively contribute to the physiological functions of the human body. The intricacies of its composition are profoundly influenced by dietary preferences, where the quality, quantity, and frequency of food consumption play a pivotal role in either fostering or impeding specific bacterial strains. Type 2 diabetes mellitus (T2DM) is a prevalent and deleterious condition that originates from excessive hyperglycemia. Do lifestyle interventions targeting dietary adjustments, nutritional supplements, physical activity, and weight management programs exhibit a significant relationship in altering the composition of the gut microbiome and managing T2DM? This paper aims to evaluate the effects of lifestyle interventions on patients with T2DM and the implications of these changes on disease outcomes and progression. Lifestyle interventions can significantly impact the management of T2DM, especially those targeting dietary adjustments, nutritional supplements, physical activity, and weight management programs. The adoption of a high-fiber diet and increased fruit consumption have shown positive impacts on both insulin sensitivity and the composition of the gut microbiota. Additionally, promising outcomes emerge from supplementing with Omega-3 fatty acids, Vitamin K2 (MK-7), and transglucosidase, which influence insulin levels, glycemic control, and gut microbiota composition. Personalized diet interventions and the transformative effects of the Mediterranean diet present positive outcomes in metabolic control. The intensity of exercise plays a pivotal role in shaping the composition of the gut microbiota, with moderate-intensity continuous exercise displaying positive effects on anti-inflammatory microbes. Chronic exercise showcases favorable impacts on glycemic control and systemic inflammation. Emphasizing the intricate relationship between dietary habits, gut microbiota, and the risk of T2DM underscores the potential of the gut microbiota as a universal biomarker for assessing diabetes risk. Nutritional supplements and exercise interventions provide potential avenues for the management of T2DM, emphasizing the necessity for tailored strategies. Further research is encouraged to delve into the long-term effects and intricate interplay between lifestyle factors and the gut microbiome, enhancing our understanding of T2DM pathophysiology for targeted therapeutic approaches.

## Introduction and background

Type 2 diabetes mellitus (T2DM) stands out as a prevalent metabolic disorder globally. As reported by the International Diabetes Federation in 2019, diabetes resulted in 4.2 million fatalities, and 463 million adults aged 20 to 79 were affected by diabetes-a figure anticipated to increase to 700 million by 2045 [1]. Its onset results from the interplay of two key elements: compromised insulin secretion by pancreatic β-cells and the diminished responsiveness of insulin-sensitive tissues to insulin [1]. According to the WHO, diabetes mellitus is a persistent metabolic condition marked by increased blood glucose levels, eventually causing damage to the heart, blood vessels, eyes, kidneys, and nerves [1]. The human gut, hosting approximately 100 trillion microbes across 5000 species, contains ten times more microbial cells than the entire human body. These microbes play a crucial role in influencing energy harvesting, maintaining a balance in microbial composition, and producing essential substances like vitamins [2]. Dietary changes significantly impact the balance in the composition of the gut microbiome between beneficial and opportunistic microbes, consequently influencing overall health [2]. *Bacteroides*, *Proteobacteria*, *Firmicutes*, and *Actinobacteria* were prominent in T2DM patients and healthy controls, but their abundance exhibited significant variations [3]. The balance between *Bacteroidetes* and *Firmicutes* is a significant factor in influencing health and disease [3]. A notable decrease in bacteria such as *Lactobacilli spp.* and *F. prausnitzii* was linked to insulin resistance, while an elevated BMI in T2DM patients correlated with heightened levels of *Akkermansia muciniphila* (*A. muciniphila*), associated with reduced fat metabolism and increased BMI [3].

The initial approach to managing type 2 diabetes involves implementing changes in diet, engaging in physical activity, and achieving weight loss, either before or simultaneously with the introduction of pharmacological treatment [4]. Diet significantly influences the composition of the gut microbiota. The Western diet pattern, characterized by high-fat and low-fiber intake, is associated with lower microbial diversity and richness. This pattern sees reductions in *Bacteroidetes* and increases in *Firmicutes* and *Proteobacteria*, which potentially contribute to diseases like obesity, diabetes, and other pro-inflammatory conditions [5]. High-fat diets elevate *Alistipes* and *Bacteroides* species while reducing *Faecalibacterium*. Conversely, omega-3 polyunsaturated fatty acids (PUFAs) promote butyrate-producing bacteria and increase *Bifidobacterium* and *Lactobacillus* [5]. Long-term consumption of animal-protein-rich diets is associated with an abundance of *Alistipes* and *Bacteroides* and decreased levels of *Roseburia*, whereas plant-based protein sources lead to higher levels of* Lactobacilli* and *Bifidobacteria*. Such diets result in a gut microbiome characterized by higher alpha diversity and increased levels of *Bacteroidetes*, *Prevotell*a, and *Roseburia* [5]. Excessive calorie consumption and insufficient physical activity, prevalent in numerous contemporary societies, are two significant contributors to the widespread obesity epidemic [6]. Regular exercise contributes to increased diversity in gut microflora, exemplified by a greater abundance of *Faecalibacterium prausnitzii* (*F. prausnitzii*) in athletes, contributing to a healthier intestinal environment [7]. Athletes and individuals with low BMI exhibit elevated levels of A. muciniphila in their microbiota compared to those with high BMI. The mucin-degrading A. muciniphila demonstrates an inverse correlation with BMI, obesity, and metabolic disorders [7].

Aims

In light of the pervasive prevalence of diabetes, exploring effective methods for its control becomes increasingly imperative. Lifestyle interventions have emerged as promising strategies, demonstrating success in managing diabetes and mitigating associated risks. This paper endeavors to evaluate the impact of these lifestyle changes on the gut microbiota, aiming to uncover potential correlations that could enhance the efficacy of diabetes treatment and management. Understanding the intricate interplay between lifestyle interventions, gut microbiota, and diabetes could pave the way for more targeted and personalized therapeutic approaches, fostering improved outcomes for individuals grappling with this widespread metabolic disorder.

## Review

Methods

A systematic and deliberate examination of the literature was carried out using three electronic databases from January 1, 2013, to December 31, 2023: PubMed, ScienceDirect, and ProQuest. The search employed keywords such as ‘type 2 diabetes and dietary intervention and gut microbiota’, ‘type 2 diabetes and lifestyle intervention and gut microbiota’, and ‘type 2 diabetes and exercise intervention and gut microbiota’. The focus was on peer-reviewed experimental and observational publications. Studies that appeared as duplicates, were not written in English, or were published prior to 2013 were excluded from the review.

Following acquisition, publications were assessed based on their title, study abstract, and full-text accessibility. The preliminary analysis of the databases yielded 93,288 publications. The abstracts' information was cross-referenced with the keywords, resulting in a specific number of publications chosen that aligned with the objective of this study. A total of 24 publications were selected in accordance with the specified criteria.

Inclusion Criteria

The selected publications were determined by the subsequent inclusion criteria: publications ranging from 2013 to 2023, conducted on human subjects, focused on gut microbiota in T2DM without significant impacts from anti-diabetic medications, peer-reviewed experimental or observational studies, and available in full text.

Exclusion Criteria

**Figure 1:**
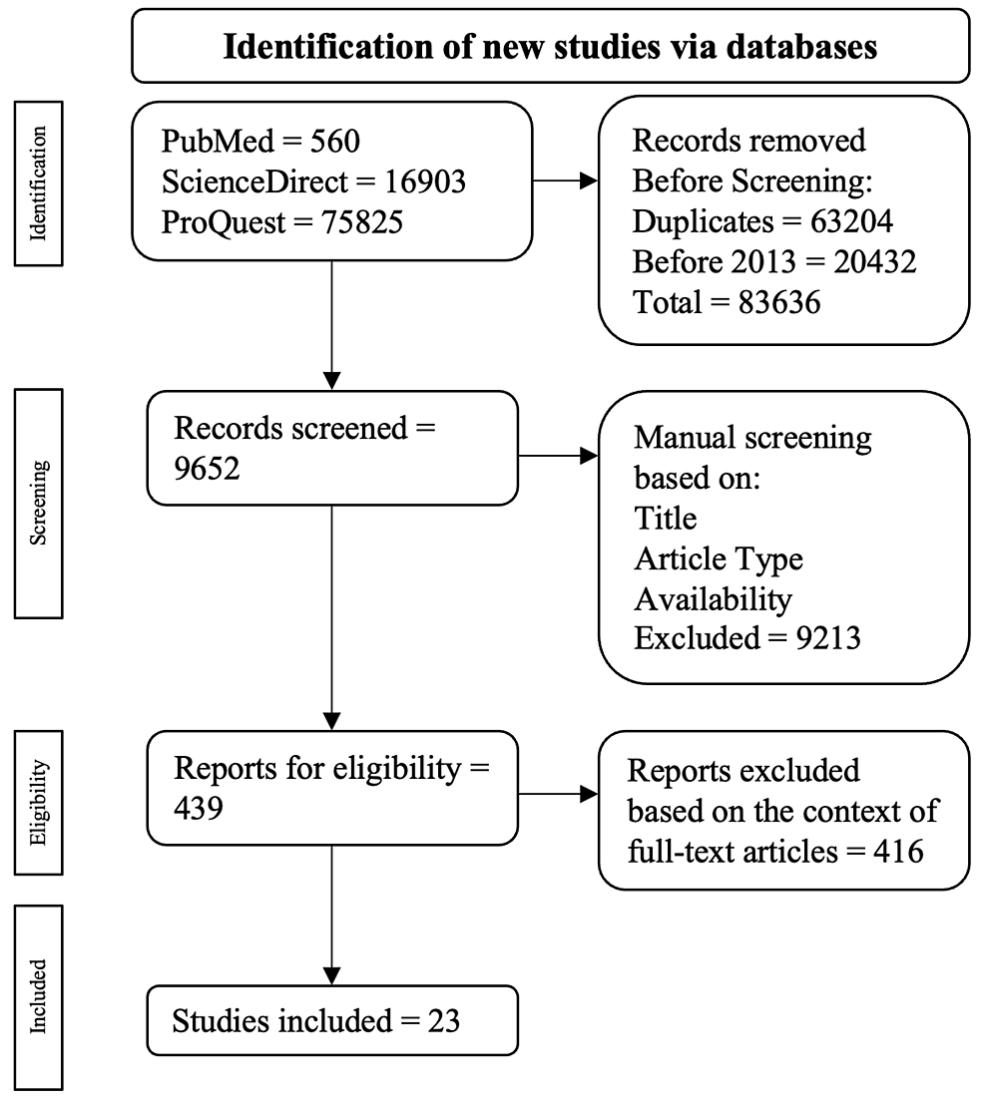
Flowchart of the PRISMA-based algorithm search employed. Source: [8] PRISMA: Preferred Reporting Items for Systematic Reviews and Meta-Analyses.

Publications were eliminated according to the subsequent exclusion criteria: replicated publications, articles not in English, and publications lacking full text. The process of selecting articles through inclusion and exclusion criteria is elaborated in Figure 1.

Bias

Each publication was evaluated for bias using the Grading of Recommendation, Assessment, Development, and Evaluation (GRADE) scale, yielding a moderate bias rating. The GRADE tool was selected for its comprehensive consideration of errors in publications, encompassing aspects such as publication bias, indirectness, and imprecision.

Results

A total of 93,288 publications were identified: 560 originated from PubMed, 16,903 from ScienceDirect, and 75,825 from ProQuest. Among these, 63,204 were redundant publications, and 20,432 were published prior to 2013. Consequently, 9,652 publications were eliminated during the automated screening process, leaving 83,636 publications for manual review. These publications were subsequently subjected to manual assessment based on their full-text accessibility, title, and study type, resulting in 415 publications undergoing thorough eligibility verification through full-text analysis. Ultimately, a curated selection of 24 publications was made.

Examining the daily food intake of individuals with T2DM reveals profound implications for their health. Strong associations were identified between dietary habits, gut microbial composition, and T2DM risk. Obese individuals exhibited an elevated T2DM risk, with a notable decrease in *Prevotella* and an increase in *F. prausnitzii* and lactic acid bacteria among non-obese individuals, promoting a healthier gut microbiome. The positive impact of a high-fiber diet on glucose homeostasis was evident through reductions in HbA1c levels, fasting blood glucose levels, and inflammatory cytokines. Increased fruit consumption, rich in fiber, was pivotal in enhancing insulin sensitivity, decreasing HbA1c levels, and positively influencing gut microbiota composition. Omega-3 fatty acids were associated with heightened gut microbiota diversity, enhanced insulin sensitivity, and decreased inflammatory markers. The intensity of exercise significantly impacted gut microbiome composition and metabolic function. Moderate-intensity continuous exercise increased beneficial microbes, while high-intensity continuous exercise potentially reduced pathogenicity. Chronic exercise positively affected functional and anthropometric variables, glycemic control, and systemic inflammation. All synthesized information is found in Table 1 [9-31].

**Table 1:** Summarization of all articles included in this review according to PRISMA guidelines. Source: [8] T2DM: Type 2 Diabetes Mellitus; FBG: Fasting Blood Glucose; EGCG: Epigallocatechin-3-Gallate; TCM: Traditional Chinese Medicine; DHA: Docosahexaenoic Acid; EPA: Eicosapentaenoic Acid; BCAA: Branched Chain Amino Acids; GLP-1: Glucagon Like Peptide 1; ImP: Imidazole Propionate; MAPK: Mitogen-Activated Protein Kinase; SCFA: Short-Chain Fatty Acid; HOMA-IR: Homeostatic Model Assessment for Insulin Resistance; VLDL: Very Low Density Lipoprotein; ROS: Reactive Oxygen Species; MICT: Moderate-Intensity Continuous Training; PRISMA: Preferred Reporting Items for Systematic reviews and Meta-Analyses.

S. No.	Author	Country	Design & Study Population	Findings	Conclusion
1	Díaz-Rizzolo DA et al. (2020) [9]	Spain	Cross-Sectional Study (n = 182)	Obese individuals were at a higher risk of developing T2DM and had worse overall metabolic outcomes. By comparing the gut microbiota between obese and non-obese patients, it was shown that there was a significant decrease in Prevotella in the non-obese patients. There was an overall increase in Faecalibacterium prausnitzii and lactic acid bacteria, both promoting a healthy gut biome.	This study concluded that having a healthy diet and not being obese will positively contribute to the overall health of the gut microbiome. This study also showed that having a healthy diet and not being obese will help protect against developing T2DM.
2	Chen L et al. (2023) [10]	China	Randomized Controlled Trial (n = 9)	A high-fiber diet has been shown to improve glucose homeostasis in patients with T2DM. A high-fiber diet significantly decreased the serum levels of HbA1c and FBG. Mutating the gut microbiota by adding a high-fiber diet resulted in lower levels of inflammatory cytokines and increased tight junction integrity. High fiber diets resulted in increased levels of Firmicutes and Bacteroidota levels, resulting in better insulin control.	Supplementation with a high-fiber diet lowered HbA1c and FBG and increased the gut microbiota diversity in patients with T2DM.
3	Jiang Z et al. (2020) [11]	China	Randomized Controlled Trial (n = 4084)	Increased consumption of fruits and vegetables resulted in an increased level of Faecalibacterium, Akkermansia, Clostridiales, and Acidaminoccus. Increased fruit and vegetable consumption was inversely associated with Fusobacterium, which is positively associated with T2DM.	It has been shown that increasing one's diet with fruits and vegetables directly correlates to increased gut microbiota diversity. This increased diversity has been shown to decrease T2DM.
4	Ito A et al. (2023) [12]	Japan	Observational Study (n = 91)	Daily green tea consumption, particularly in seasonal variations, plays a pivotal role in maintaining fasting blood glucose and insulin levels, with P. vulgatus identified as a potential mediator in the negative association between green tea intake and fasting blood glucose levels. Green tea may improve glucose metabolism by suppressing the abundance of P. vulgatus, which is linked to elevated blood glucose levels in individuals without T2DM.	This four-season observational study revealed that habitual green tea intake, rather than isolated catechins or EGCG, was significantly associated with improved glucose metabolism and alterations in the relative abundances of specific gut microbial species in individuals without T2DM.
5	Hu B et al. (2023) [13]	China	Randomized Controlled Trial (n = 45)	The addition of Maccog TCM tea in patients with T2DM resulted in a statistically significant decrease in fasting plasma glucose levels and total lipid levels. Supplementation with Maccog TCM resulted in increased levels of Clostridial and Lactobacillus. Maccog TCM supplementation resulted in increased overall gut microbiota diversity, resulting in an improvement in glucose metabolism.	Supplementation with Maccog TCM tea decreased FBG and cholesterol levels in patients with T2DM.
6	Luo W et al., (2022) [15]	China	Experimental Study (n = 33)	A diet strategy of medicine-derived food plants and whole grains accompanied by intermittent energy restriction effectively reverses high blood sugar in patients with T2DM by enhancing pancreas function, reducing inflammation in the pancreas, increasing insulin production, and significantly increasing the abundance of Bacteroidetes, Parabacteroides, and Roseburia.	Chinese medical nutrition therapy with medicine food homologous plants may be a promising nutritional intervention in patients with T2DM
7	Mateo-Gallego R et al., (2021) [16]	Spain	Experimental Study (n = 14)	Consumption of alcohol-free with modified composition led to a significant increase in the abundance of Parabacteroides, compared to a regular alcohol-free beer. It is suggested that this compound could act as a prebiotic for Parabacteroides, favoring its growth.	The alteration of the gut microbiota due to alcohol-free beer may explain the improvement seen in insulin resistance
8	Karusheva Y et al., (2019) [17]	Germany	Crossover Double-Blinded (n =12)	Short-term dietary reduction of BCAAs in individuals with T2DM led to decreased insulin secretion, increased postprandial insulin sensitivity, enhanced mitochondrial efficiency in adipose tissue, and altered gut microbiome composition favoring Bacteroidetes.	The study concludes that a brief reduction in dietary BCAAs acutely impacts insulin dynamics, postprandial insulin sensitivity, and mitochondrial efficiency in adipose tissue while also influencing gut microbiota composition.
9	Ren M et al., (2020) [18]	China	Experimental Study (n = 45)	A low carbohydrate diet significantly improved HbA1c and depression while significantly increasing bacteria that produce short-chain fatty acids, such as Ruminococcus, Roseburia, and Eubacterium, and increasing GLP-1 concentration	A low-carb diet can improve depression, which may be associated with the increased growth of bacteria that produce short-chain fatty acids.
10	Molinaro A et al., (2020) [19]	Sweden	Population Study (n = 1,990)	ImP levels were increased in patients with T2DM, regardless of their country of origin, and this was associated with a low abundance of microbial diversity and a specific gut microbiome enterotype. An unhealthy diet, but not histidine intake, was associated with increased ImP levels, suggesting that an unhealthy diet can change the microbial environment in the gut and increase the production of ImP.	An unhealthy diet may contribute to an altered microbial community type with increased potential to metabolize dietary histidine to ImP. This contributes to impaired glucose metabolism by activating MAPK signaling, leading to the degradation of insulin receptor substrate and inflammatory signaling.
11	Ismael S et al., (2021) [20]	Portugal	Experimental Study (n = 9)	The Mediterranean diet has positively impacted the metabolic control of people with T2DM. It leads to a decrease in HbA1c levels, associated with a decrease in microvascular complications. Additionally, adherence to the Mediterranean diet increased gut microbiota diversity and richness, which promote gut homeostasis and have been linked to improvements in glucose metabolism and insulin secretion.	The Mediterranean diet effectively improves metabolic control of type 2 diabetes patients, and changes in gut microbiota may precede changes in standard biomarkers of type 2 diabetes.
12	Yamaguchi Y et al., (2016) [21]	Japan	Case-Control Study (n = 59)	A high carbohydrate, fat, and protein consumption was positively associated with a higher count of Clostridium clusters IV and XI and a lower Bifidobacterium spp, Clostridium cluster IV, and order Lactobacillales. Protein consumption was negatively correlated with total SCFAs and fecal acetate. Insulin levels and HOMA-IR were negatively correlated with propionate, total SCFAs, and acetate.	Low protein and carbohydrate diets support a healthy gut microbiome and improved glucose tolerance in T2DM.
13	Wang H et al., (2022) [22]	China	Cohort study (n = 2772)	Specific gut microbial genera were associated with glycemic traits and altered the risk of T2DM, with some microbial genera showing consistent associations across regions. Moreover, it was noted that dietary and lifestyle factors also held a link in these microbial genera variety.	This study concludes that gut microbiota composition varies across varying regions of China and is influenced by dietary factors.
14	Meleshko T et al., (2021) [23]	Ukraine	Randomized Control Trial (n = 56)	The study indicates that a personalized diet intervention for patients with T2DM leads to significant improvements in various biochemical markers, including VLDL, glucose, and uric acid, coupled with notable changes in gut and oral microbiota such as Entercoccus faecalis, Escherichia coli, Lactobacillus, and Candida, contributing to positive alterations in physical parameters such as body weight and circumferences.	The observed improvements in biochemical, immunological, and microbiota parameters and positive changes in physical parameters support the feasibility and efficacy of a personalized diet as a potential therapeutic approach for managing T2DM.
15	Kumar M et al., (2022 [24]	Spain	Cohort Study (n = 35)	At the end of this study, there was no significant change in total body mass index between the control and experimental groups. No statistically significant differences were observed in the total gut microbiota composition between the two groups. There was a significant increase in the proportion of Bacteroides to Prevotella in the experimental group. There was a decreasing trend in Firmicutes/Bacteroides levels in the control group.	More studies need to be conducted to see if a longer duration of sardine consumption would alter the glycemic index in patients with T2DM. However, for this study, it was deduced that 100g of sardines 5 days a week does not improve glycemic control. Still, increased DHA and EPA levels could benefit the cardiovascular system.
16	Kumar M et al., (2022) [24]	India	Peer-Review	Increased consumption of Omega-3 fatty acids in patients with T2DM has been linked to increased overall gut microbiota diversity. Increased levels of Omega-3 fatty acids have been associated with decreased production of ROS. Increased consumption of Omega-3 fatty acids decreased insulin resistance and improved mitochondrial B-oxidization of fatty acids.	Omega-3 supplementation has been shown to increase gut diversity in patients with T2DM, decrease ROS production, and increase insulin sensitivity.
17	Kumar M et al., (2022) [24]	Japan	Experimental Study (n = 60)	There is a significant decrease in Clostridium cluster IV and a significant increase in Bifidobacterium and Lactobacillales in T2DM patients compared to healthy individuals. In the transglucosidase group, there was a significant increase in the Bacteroidetes-to-firmicutes ratio.	Transglucosidase decreases blood glucose levels, preventing weight gain in TD2M patients and modulating gut microbiota.
18	Zhang Y et al., (2023) [25]	China	Randomized Controlled Trial (n = 60)	Vitamin K2 (MK-7) supplementation is associated with significantly decreased insulin levels, fasting glucose, and HbA1c levels in individuals with T2DM compared to controls. Higher concentrations of secondary bile and short-chain fatty acids were found in the vitamin K2 supplementation group, along with increased richness of the genera biosynthesizing these metabolites.	Vitamin K2 may play a key role in regulating glycemic homeostasis in patients with T2DM and thus may have clinically beneficial applications in treating this patient population.
19	Torquati L et al., (2023) [27]	UK	Prospective Randomized Controlled Trial (n = 12)	Alpha diversity and relative abundance of specific microbial taxa significantly differed between the participants who did 8 weeks of combined aerobic and resistance moderate-intensity continuous training compared to those who did the high-intensity continuous training.	Exercise intensity significantly affects abundance within the gut microbiome and metabolic function while having negligible effects on short-chain fatty acids and output.
20	Motiani KK et al., (2020) [28]	Finland	Randomized Control Trial (n = 26)	Short-term exercise training, particularly MICT, demonstrates the potential to modulate gut microbiota composition, decrease the Firmicutes/Bacteroidetes ratio, and alleviate markers of intestinal inflammation.	The study reveals that exercise training for a short duration significantly reduces intestinal inflammation, decreases endotoxemia, and induces favorable alterations in gut microbiota profiles in insulin-resistant individuals.
21	Pasini e et al., (2019) [29]	Italy	Experimental Study (n = 30)	T2DM leads to a significant overgrowth of intestinal mycetes, systemic low-grade inflammation, and increased intestinal permeability. Exercise improved functional and anthropometric variables and glycemia. Chronic exercise reduces leaky gut, intestinal mycetes overgrowth, and systemic inflammation.	Exercise modifies the intestinal microbiota composition and the function of the barrier that controls diabetes.
22	Wei S et al., (2022) [30]	Denmark	Post-hoc Analysis of a Randomized Clinical Trial (n = 98)	Changes in the diversity of gut microbiota in patients with T2DM were documented at baseline, 3 months, 6 months, 9 months, and 12 months, and no significant statistical differences were detected. Furthermore, the changes in the diversity of gut microbiota did not correlate with the clinical changes observed in the patients in the treatment group, nor did they mediate said changes.	In patients with T2DM, changes in the composition of gut microbiota are unlikely to explicate the clinical benefits or enhancements of pharmacological or intensive lifestyle interventions.
23	Frost F et al., (2019) [31]	Qatar	Experimental Study (n = 26)	Following a structured low-calorie formula diet for 6 weeks, followed by a 9-week food reintroduction period, can greatly increase the gut microbiota in patients. All patients had a significant decrease in HbA1c, fasting glucose, and insulin levels by the end of the trial. There was a significant decrease in levels of Collinesella in all patients, which indicated lower levels of atherosclerosis and plaque buildup.	This study showed that a structured weight loss program can positively impact the gut microbiota in patients with T2DM. This study also showed that this structured weight loss program can have possible cardiovascular benefits in addition to increasing the gut microbiota.

Discussion

Dietary Interventions

The diverse array of dietary interventions explored in the reviewed studies offers valuable insights into the intricate relationship between diet and T2DM. The daily food intake of individuals with T2DM significantly impacts their health and well-being through various mechanisms, as evidenced by the studies included in this review. Strong associations between dietary habits, gut microbial composition, and T2DM exist. It was found that obese individuals have an elevated risk of developing T2DM and experiencing poorer metabolic outcomes [9]. A comparison of gut microbiota in obese and non-obese patients demonstrated a noteworthy decrease in *Prevotella*, alongside an overall increase in *F. prausnitzii* and lactic acid bacteria among non-obese individuals. The latter two have been found to promote a healthy gut microbiome [9]. Maintaining a healthy diet and avoiding obesity emerged as positive contributors to overall gut microbiome health and offered protection against the development of T2DM [9]. Insulin resistance, dyslipidemia, and fatty liver, associated with T2DM, exhibit a higher prevalence in individuals with low bacterial richness in the gut microbiota than in those with high bacterial richness [9]. Gut environments characterized by low bacterial richness showed higher species dominance from the genus *Bacteroides*, while high bacterial richness was significantly associated with *Bifidobacterium*, *Lactobacillus*, and *Akkermansia* [9].

The positive influence of a high-fiber diet on glucose homeostasis is underscored by reductions in HbA1c levels, fasting blood glucose levels, and inflammatory cytokines [10]. Moreover, a high-fiber diet also led to favorable changes in the composition of gut microbiota by increasing levels of *Bifidobacterium*, thus resulting in better insulin control [10]. Similarly, increased fruit consumption, which is also high in fiber, was found to play a pivotal role in enhancing insulin sensitivity and reducing HbA1c levels, intricately linking diet, glycemic control, and microbiota [11]. Increased consumption of fruits resulted in an increased level of* Faecalibacterium*, *Akkermansia*, *Clostridiale*s, and *Acidaminococcus*, which are known to enhance short-chain fatty acid synthesis, maintain intestinal mucosal integrity, improve insulin sensitivity, and exhibit anti-inflammatory properties [10, 11]. Conversely, levels of Fusobacterium, which is positively correlated with T2DM, ulcerative colitis, and colorectal cancer, were reduced [11].

An association was elucidated between daily consumption of green tea and catechins, gut microbiota, and glucose metabolism, revealing a negative correlation between green tea and catechin intake with insulin levels and fasting blood glucose [12]. *Phocaeicola vulgatus* (*P. vulgatus*) was identified as a potential mediator in the negative association between green tea intake and fasting blood glucose levels. Green tea may improve glucose metabolism by suppressing the abundance of *P. vulgatus*, which has been linked to elevated blood glucose levels in individuals without T2DM [12]. Similarly, the dietary inclusion of Traditional Chinese Medicine Tea, with mulberry leaf, radix astragali, corn stigma, cortex lycii, radix ophiopogonis, and gynostemma, called “maccog” tea for 12 weeks, resulted in decreased levels of fasting blood glucose and cholesterol in patients with T2DM, while also increasing ov

Evaluating alternative protein sources, such as sardines, Balfegó M et al. revealed that although consuming 100g of sardines five times per week failed to improve glycemic control, they exhibited potential cardiovascular benefits, emphasizing the nuanced impact of specific dietary components [14]. The incorporation of whole grains and periods of fasting into the diet increases the abundance of *Bacteroides*, which promotes the production of short-chain fatty acids, thus yielding improvement in HbA1c levels and pancreatic islet β-cell function by promoting the secretion of GLP-1 [15]. Changes in the abundance of *Faecalibacterium* were negatively associated with bread intake and positively associated with rice intake, while changes in the abundance of *Akkermansia* were negatively associated with potato intake [15]. In addition to incorporating whole grains and protein into the participants’ daily diet, the consumption of alcohol-free beer was also analyzed. Alcohol-free beer, modified by eliminating maltose and adding isomaltulose and resistant maltodextrin, demonstrated improvement in insulin resistance in subjects with T2DM who were overweight or obese and drank alcohol-free beer [16]. These promising results may be partially mediated by the gut microbiota, particularly by a member of the *Porphyromonadaceae* family, the genus *Parabacteroides* [16]. These findings provide further evidence that dietary intervention, by adding nutrients, can significantly affect the gut microbiome in humans and could be an important mechanism responsible for diet-induced cardiometabolic changes [16].

Studies have also evaluated the effects of reducing certain dietary components, yielding many interesting findings across the reviewed studies. Increased concentrations of branched-chain amino acids in the plasma are known to be predictive markers for impaired insulin signaling [17]. As such, short-term reduction of dietary branched-chain amino acids led to improved postprandial insulin sensitivity, decreased postprandial insulin secretion, and an increased abundance of intestinal *Bacteroidetes* in patients with T2DM [17]. Compared to a low-fat diet, a low-carbohydrate diet in patients with T2DM improved HbA1c and depression, while also increasing GLP-1 concentrations. These results may be associated with the increased growth of bacteria that produce short-chain fatty acids, such as *Ruminococcus* and *Roseburia* [18]. The risk of T2DM may be linked to dietary habits, as evidenced by apparent associations between elevated serum imidazole propionate (ImP) levels, pro-inflammatory microbiota, and impaired glucose metabolism [19]. Furthermore, an unhealthy diet, especially one low in fiber, may contribute to an altered microbial community type with increased potential to metabolize dietary histidine to ImP, which in turn contributes to impaired glucose metabolism by activating MAPK signaling, leading to the degradation of insulin receptor substrate and inflammatory signaling [19].

The transformative effects of the Mediterranean diet were explored, consistently demonstrating positive impacts on metabolic control in people with T2DM [20]. Following the Mediterranean diet entails increased intake of plant-based foods, moderate consumption of meat products, substituting herbs and spices for salt, and reliance on olive oil as the primary fat source [20]. Adherence to the Mediterranean diet led to a decrease in HbA1c levels, which is associated with a decrease in microvascular complications [20]. Additionally, adherence to the Mediterranean diet increased gut microbiota diversity and richness, showcasing an increase in the *Prevotella:Bacteroides* ratio, promoting gut homeostasis, and improving glucose metabolism and insulin secretion [20]. Low-protein and low-carbohydrate diets support a healthy gut microbiome as well as improved glucose tolerance in T2DM. High levels of carbohydrate, fat, and protein consumption were found to be positively associated with a higher abundance of *Clostridium* and *Bacteroides* and a lower abundance of *Bifidobacterium* [21].

The intricate relationship between dietary habits, gut microbiota, and T2DM risk was emphasized through the creation of a novel healthy microbiome index (HMI) by Wang H et al. The study introduced an HMI that was associated with T2DM risk across different geographic regions, age groups, sexes, BMI levels, and urbanization levels, highlighting the potential of gut microbiota as a universal biomarker for diabetes risk assessment and the significance of dietary intervention in diabetes as an important aspect of effective management [22]. A personalized diet intervention for patients with T2DM led to significant improvements in various biochemical markers, including very-low density lipoprotein (VLDL), glucose, creatinine, urea, magnesium, sodium, and uric acid, coupled with notable changes in both gut and oral microbiota. These contributed to positive alterations in physical parameters such as body weight and circumference [23]. These findings support the feasibility and efficacy of personalized diets as potential therapeutic approaches for managing T2DM and underscore the importance of considering individualized dietary plans in diabetes treatment [23]. Increasing gut microbiota diversity was a key factor associated with improved glucose metabolism [10, 21]. Specific microbial genera, including *Bifidobacterium*, *Faecalibacterium*, *Akkermansia*, and *Lactobacillus*, were implicated in positive metabolic outcomes across studies [10, 21]. Higher microbial diversity, as seen with increased intake of fruits, vegetables, and certain dietary patterns, was consistently associated with improved glycemic control and a decreased risk and/or progression of T2DM [10, 21].

Nutritional Supplements

Exploring Omega-3 fatty acid, Vitamin K2 (MK-7), and transglucosidase supplementation has revealed promising outcomes in influencing insulin levels, glycemic control, and gut microbiota composition. Specifically, Omega-3 fatty acids have been associated with heightened gut microbiota diversity, increased abundance of butyrate-producing microbes such as *Bacteroides* and *Roseburia*, along with enhanced insulin sensitivity, improved mitochondrial β-oxidation of fatty acids, decreased production of reactive oxygen species (ROS), decreased insulin resistance, and decreased inflammatory markers [24]. Supplementation with vitamin K2 (MK-7) is associated with significantly decreased insulin levels, fasting glucose, and HbA1c levels in individuals with T2DM compared to controls. Increased levels of circulating MK-7 were found to be significantly associated with a lower *Firmicutes:Bacteroidetes* ratio [25]. Higher concentrations of secondary bile and short-chain fatty acids were found in the vitamin K2 (MK-7) supplementation group, along with increased richness of the genera biosynthesizing these metabolites [25]. Metabolic diseases such as T2DM may lead to a decreased relative abundance of microbes in the gut that produce vitamin K2, and supplementing vitamin K2 in this patient population may improve glycemic control and patient treatment outcomes [25]. Transglucosidase supplementation decreases blood glucose levels and prevents weight gain in patients with T2DM by stimulating oligosaccharide production in the alimentary tract, alongside increasing abundances of *Lactobacillus* and *Bifidobacterium* and increasing the ratio of *Bacteroidetes:Firmicutes* [26].

Exercise and Physical Activity

While dietary modifications are crucial, research analyzing the effects of exercise on the gut microbiota is also important to consider. In people with T2DM who were minimally active at baseline, exercise intensity (moderate versus high intensity) was found to have significant effects on the relative abundances within the gut microbiome and metabolic function, while having negligible effects on short-chain fatty acids and output [27]. Moderate-intensity continuous exercise engendered an increased abundance of *Bifidobacterium* and *Akkermansia*, known to produce anti-inflammatory acetate and butyrate, whereas high-intensity continuous exercise was found to potentially play a role in reducing the pathogenicity of gut microbes [27]. Exercise interventions, including both sprint interval training (SIT) and moderate-intensity continuous training (MICT), were found to significantly improve HbA1c and reduce the systemic inflammatory marker TNF-α [28]. Short-term exercise training, particularly MICT, demonstrates the potential to modulate gut microbiota composition by decreasing the *Firmicutes:Bacteroidetes* ratio, alleviating markers of intestinal inflammation, and improving insulin sensitivity. This highlights exercise as a promising intervention to mitigate the risk of various diseases associated with obesity and insulin resistance, such as T2DM [28]. T2DM can lead to systemic low-grade inflammation and increased intestinal permeability, which can be reduced or improved through chronic exercise [29]. Exercise has been found to improve functional and anthropometric variables, enhance glycemic control, decrease cholesterol, and decrease systemic inflammation (measured as c-reactive protein (CRP)) in patients with T2DM [29].

While many studies tout the benefits of exercise as an effective synergistic tool in the management of T2DM, the positive effects observed in T2DM management through intensive lifestyle interventions, including a combined exercise and diet regimen, were not found to be attributable to, or directly caused by, alterations in the composition of the gut microbiota, according to Wei S et al. [30]. A structured weight loss program can positively influence the gut microbiome in individuals with T2DM by fostering increased diversity and concurrently diminishing the prevalence of inflammatory microbes like *Collinsella* [31]. This reduction hints at potential cardiovascular advantages, marked by lower levels of atherosclerosis and plaque buildup [31]. Simultaneously, there is an elevation in the abundance of beneficial microbes such as *Bacteroides* and *Faecalibacterium*, contributing to the overall well-being of the gut microbiome and protecting the patient from dysbiosis [31]. While the effect of exercise on the gut microbiome, especially in patients with T2DM, is a topic of research interest, it is just one aspect of the complex interplay between the gut microbiota, diet, genetics, and various health conditions, including diabetes. More research is needed to fully understand the implications of combining all these factors in the context of T2DM

Strengths and limitations

The strengths of this study include the exploration of diverse interventions and consideration of various outcome measures, providing a comprehensive overview. Additionally, the reliability is enhanced by multiple studies supporting each intervention. The limitations of this study involve the variability in study designs and participant characteristics across studies, inherent to systematic reviews like this one. Furthermore, the limited long-term follow-up in some interventions restricts our ability to fully understand the scope and ramifications of these interventions over time.

## Conclusions

This systematic review offers a comprehensive synthesis of evidence supporting the role of lifestyle interventions in the management of T2DM, focusing on dietary modifications, nutritional supplements, physical activity, and weight management programs. The common themes identified here present valuable insights for clinicians, researchers, and patients seeking strategies for effective diabetes care. The findings suggest promising avenues for managing T2DM through lifestyle interventions. Incorporating these strategies into clinical practice and public health initiatives may improve patient outcomes and reduce healthcare burdens. This review contributes valuable insights into the multifaceted interventions impacting T2DM, emphasizing the need for tailored and comprehensive strategies. The collective findings highlight the potential of personalized interventions, emphasizing the importance of tailored dietary plans and exercise regimens in T2DM management. Future research should investigate the long-term effects of these interventions and their sustainability. Moreover, examining the interplay between lifestyle factors and the gut microbiome could reveal novel therapeutic targets and enhance our understanding of T2DM pathophysiology.
